# Rootstock Priming with Shikimic Acid and *Streptomyces griseus* for Growth, Productivity, Physio-Biochemical, and Anatomical Characterisation of Tomato Grown under Cold Stress

**DOI:** 10.3390/plants11212822

**Published:** 2022-10-24

**Authors:** Eman G. Sayed, Abdel Wahab M. Mahmoud, Ahmed Abdel-Wahab, Reham M. El-bahbohy, Samah N. Azoz

**Affiliations:** 1 Department of Vegetable Crops, Faculty of Agriculture, Cairo University, Giza 12613, Egypt; 2 Department of Agricultural Botany, Faculty of Agriculture, Cairo University, Giza 12613, Egypt

**Keywords:** *Solanum* *lycopersicum* L., cold stress, grafting, seed priming, *Streptomyces griseus*, shikimic acid

## Abstract

With this research, we aimed to determine the impact of grafting and rootstock seed treated with *Streptomyces griseus* (*MT210913)* (*S. griseus*) or shikimic acid (SA) at a 60 ppm concentration on tomato (*Solanum lycopersicum* L.) production grown under low-temperature conditions. Two open-field trials were performed during both winter seasons of 2020 and 2021 at the Experimental Farm, Faculty of Agriculture, Cairo University, Giza, Egypt. A tomato cultivar (Peto 86) was used as a scion and two tomato phenotypes were employed as rootstocks (*Solanum cheesmaniae* L*. (line LA 524)* and GS hybrid), as well as self-grafted as a control. Effects of sub-optimal temperature on vegetative growth, yield, and fruit quality were tested. The results indicate that, under cold stress, rootstock seed priming, especially with *S. griseus*, enhanced plant growth, total yield, and fruit quality properties. GS hybrid rootstock was more effective than that of *S. cheesmaniae* rootstock in terms of mitigating the negative effect of cold stress. GS hybrid, inoculated with *S. griseus,* increased the total yield per plant by 10.5% and 5.7% in the first and second seasons, respectively. Higher levels of GA3 and mineral content were noticed in leaves that were grafted and treated with *S. griseus* compared to the control treatment. Additionally, the great enhancing effects of all anatomical features of tomato plants were recorded with GS hybrid rootstock, inoculated by *S. griseus*. These results prove that grafting on GS hybrid rootstock treated with *S. griseus* is a potential choice to alleviate the cold stress of commercial tomato varieties.

## 1. Introduction

Tomato fruits (*Solanum lycopersicum* L.) are one of the most popular and frequently consumed vegetable crops worldwide. A commercial variety success in Egypt depends on a high-quality yield. Tomato fruits are globally consumed with a production of more than 186.8 million tons per year from 5.0305 million cultivated hectares. China, India, Turkey, and the United States are the top tomatoes producers and Egypt ranks fifth [1]

Tomato growth is affected by cold stress causing shorter internodes, which limit plant height, reduce leaf expansion, decrease leaf number and total fresh weight, and increase the thickness of leaves [2]. Cold stress has an impact on root-to-shoot hormone signalling resulting in decreased plant productivity [3]. Additionally, low temperatures inhibit cell membrane fluidity as well as raise permeability [4], inhibit intra- and extracellular water and nutrient movement [5], generate reactive oxygen species (ROS) [6], might restrict photosynthesis [7], and reduce yield. 

Grafting has been employed to increase resistance to soil diseases in Japan since the late 1920s [8]. Thus, grafting has become widely used all over the world. Grafting has recently been suggested as a practical strategy to increase resistance to abiotic stress and may be an effective way to minimise or eliminate production losses [9]. Grafting superior tomato hybrids onto rootstocks is an alternate method to increase the tolerance of low temperature. These rootstocks may be inter-specific hybrids of *S. lycopersicum* with accessions of the cold tolerance of 16 wild tomato varieties [10], such as *S. ochranthum, S. cheesmaniae, S. chmielewskii, S. corneli- omulleri, S. arca-num, S. habrochaites, and S. huaylasense* [11]. Low-temperature tolerance is a complicated secondary feature that depends on numerous important attributes that are activated in roots and shoots, such as root and leaf anatomy, plant hormones, ROS scavenging enzymes, etc. Therefore, the effectiveness of a grafted plant under cold stress conditions is special to each rootstock–scion combination. Grafting onto rootstocks, which influence plant growth and cold hardiness, is thus a promising method [3].

Seed priming is an efficient, practical, and simple strategy to improve seedling vigour and yield under cold stress conditions [12,13]. Bio-priming is an environmental method that includes biological and physiological elements, such as seed hydration and inoculation with beneficial organisms to protect it against disease, that improve seed quality, enhance seedling vigour [14], increase productivity, and resist biotic and abiotic stress [15,16]. One of the most common micro-organisms found in the soil is Streptomyces. This micro-organism is responsible for producing bioactive substances, enzymes, and vitamins [17]. Streptomyces has recently gained serious attention as a plant growth-promoting (PGP) bacterium [18]. The majority of PGP bacteria stimulate plant growth by producing phytohormones, controlling plant diseases, and/or improving nutrient uptake [19]. Streptomyces has become more important in using PGP bacteria to reduce plant stress caused by drought, salinity, nutrient deficiency, metal toxicity, heat, osmotic stress, and waterlogging [20,21]. 

Nowadays, seed priming with a variety of organic and inorganic compounds is useful to diminish the negative impacts of stress conditions. The recognised precursor to aromatic amino acids, L-phenylalanine and L-tyrosine, is shikimic acid. Shikimic acid has been used for numerous years on a large scale to promote growth and improve the fruit quality of crops and vegetable plants without negative effects [22,23]. Plant growth and metabolic activity are significantly regulated by phenolic compounds [24]. In the majority of plants, sucrose is the primary photosynthetic product and the primary type of carbohydrate delivered to organs [25]. A high level of total soluble sugars was positively connected with fruit pepper quality [23]. Plant phenolics are produced through the shikimic acid pathway [26]. Hence, in the present research, we focus on the effect of rootstocks primed with *S. griseus* or SA in mitigating the harmful effect of cold stress to improve growth, fruit yield, enzyme activity, and anatomical parameters of tomato plants. 

## 2. Results

### 2.1. Plant Growth Parameters

Plant growth parameters were affected by grafting and rootstocks treated with SA and *S. griseus* (Table 1 and Table 2). All treatments, except the self-grafted Peto 86 (control treatment), recorded high values of plant height, leaf number, shoot and root fresh weight, and shoot and root dry weight of tomato plants in both seasons. Rootstock seeds treated with *S. griseus* or SA increased the plant growth parameters for both seasons.

GS hybrid rootstock treated with *S. griseus* recorded the highest plant height in both seasons. Additionally, GS hybrid rootstock primed with *S. griseus* increased leaf area by 69.46% and 69.61% compared to the control treatment in the first and second seasons (Table 1). A similar trend was observed for the fresh or dry weight of shoots and roots during both seasons (Table 2). Moreover, a significantly higher leaf number was recorded for *S. cheesmaniae* rootstock colonised with *S. griseus* when compared to the untreated self-grafted Peto 86 in the first and second seasons, respectively. Despite this, *S. cheesmaniae* rootstock decreased the leaf area by 28.86% and 28.72% compared to the self-grafted Peto 86 in the first and second seasons, respectively.

### 2.2. Fruit Yield of Tomato and Its Components

**Figure 1:**
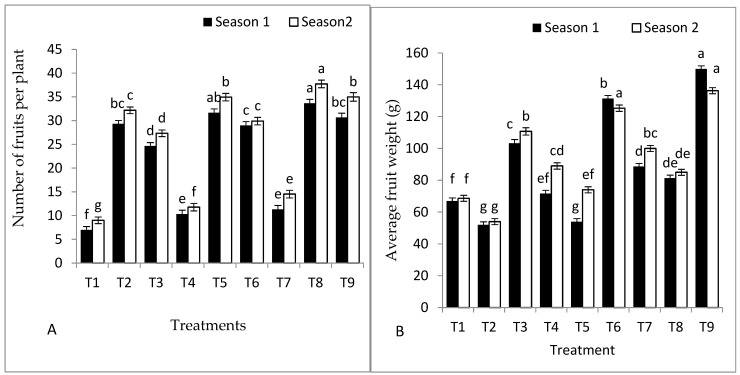

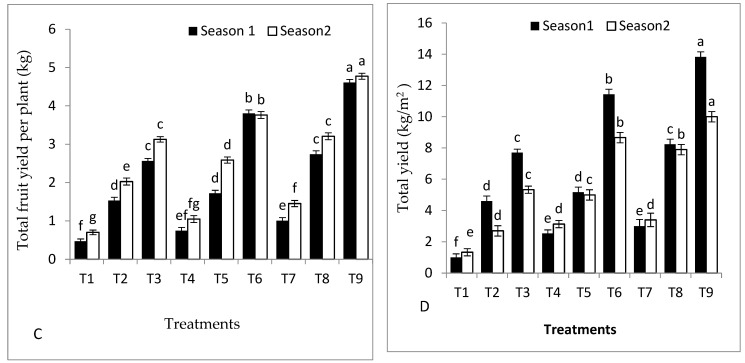
Effects of grafting, rootstock seed treated with SA, and colonisation with *S. griseus* on (**A**) average fruit weight, (**B**) number of fruits per plant, (**C**) total fruit yield per plant, and (**D**) total yield in the first and second seasons. Treatments: T1—self-grafted Peto 86/Peto 86 (control); T2—Peto 86/*S. cheesmaniae*; T3—Peto 86/GS hybrid; T4—Peto 86/Peto 86 + SA; T5—Peto 86/*S. cheesmaniae* + SA; T6—Peto 86/GS hybrid + SA; T7—Peto 86/Peto 86 + *S. griseus; T8*—Peto 86/*S. cheesmaniae* + *S. griseus*; and T9—Peto 86/GS hybrid + *S. griseus.* Standard errors of the mean are shown as vertical bars; the LSD test indicates that differences between values in each bar that are marked by different letters are significant at *p* = 0.05.

The effect of grafting, rootstock seeds primed with *S. griseus* or SA, and their combinations on yield and its components are shown in Figure 1A–D. A high fruit number per plant was recorded for *S. cheesmaniae* rootstock colonised with *S. griseus* in 2021 and 2022 seasons (Figure 1A). On the contrary, *S. cheesmaniae* rootstock recorded the lowest fruit weight compared to the untreated self-grafted Peto 86 in both seasons (Figure 1B). Moreover, GS hybrid with *S. griseus* and SA exhibited better fruit weight and fruit yield per plant during both seasons. Hence, GS hybrid, inoculated with *S. griseus* increased the fruit yield per plant by 10.5% and 5.7% in the first and second seasons, respectively. In contrast, the self-grafted Peto 86 registered the lowest fruit number and total yield per plant in both seasons (Figure 1C). Moreover, a significantly higher total yield was recorded for GS hybrid rootstock primed with *S. griseus* compared to self-grafted Peto 86 in the first and second seasons, respectively (Figure 1D).

### 2.3. Gas Exchange and Chlorophyll Measurements of Tomato Plants

**Figure 2:**
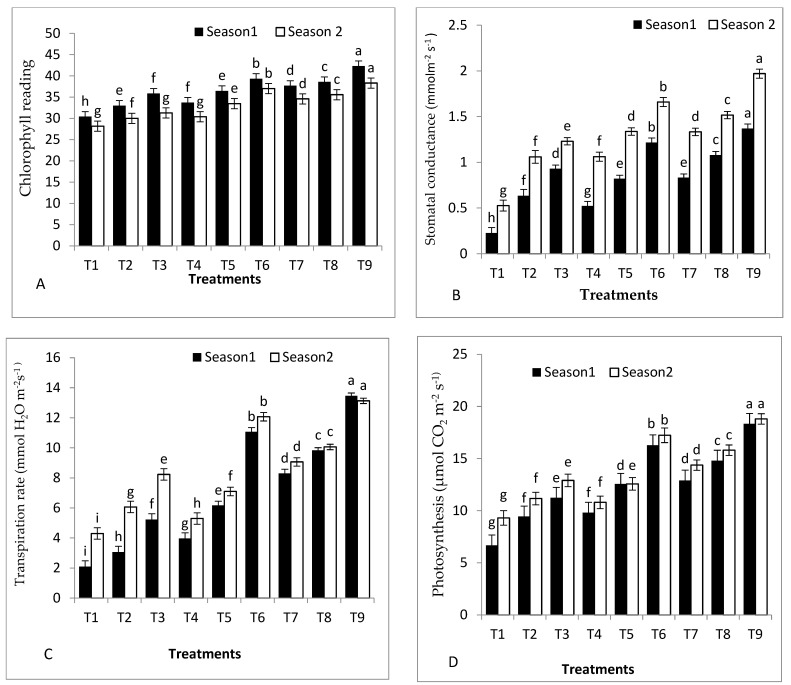
Effects of grafting, rootstock seed treated with SA, and colonisation with *S. griseus* on (**A**) chlorophyll content (SPAD), (**B**) stomatal conductance, (**C**) transpiration rate, and (**D**) photosynthesis, in the first and second seasons. Treatments: T1—self-grafted Peto 86/Peto 86 (control); T2—Peto 86/*S. cheesmaniae*; T3—Peto 86/GS hybrid; T4—Peto 86/Peto 86 + SA; T5—Peto 86/*S. cheesmaniae* + SA; T6—Peto 86/GS hybrid + SA; T7—Peto 86/Peto 86 + *S. griseus*; T8—Peto 86/*S. cheesmaniae* + *S. griseus*; and T9—Peto 86/GS hybrid + *S. griseus.* Standard errors of the mean are shown as vertical bars; the LSD test indicates that differences between values in each bar that are marked by different letters are significant at *p* = 0.05.

Chlorophyll content (SPAD), transpiration rate, photosynthesis, and stomatal conductance were significantly affected by grafting and rootstock seed treatments (Figure 2A–D). GS hybrid rootstock primed with *S. griseus* or GS hybrid rootstock primed with SA exhibited better chlorophyll content (SPAD), transpiration rate, photosynthesis, and stomatal conductance in both seasons. GS hybrid rootstock primed with *S. griseus* registered the highest chlorophyll content (SPAD), transpiration rate, photosynthesis, and stomatal conductance compared to control plants in the first and second seasons. On the contrary, the lowest values of chlorophyll content, transpiration rate, photosynthesis, and stomatal conductance were noticed with self-grafted Peto 86 in both seasons.

### 2.4. Nutrients Content in Tomato Leaves and Fruits

The results presented in Table 3 reveal that grafting treatments accumulated minerals more than the self-grafted Peto 86. It was observed that plants grafted and colonised with *S. griseus* recorded better mineral concentrations, although the self-grafted Peto 86 showed the lowest content of N, P, K, and Ca in both seasons.

Grafting and rootstock seed treatments had a significant impact on the tested Fe, Mn, and Zn concentrations in tomato leaves (Table 3). GS hybrid with *S. griseus* exhibited the highest concentration of Fe, Mn, and Zn in the first and second seasons. However, the self-grafted Peto 86 had the lowest concentrations of Fe, Mn, and Zn in both seasons.

The analysis of fruit mineral composition (Table 4) revealed that not all grafting treatments showed significant differences, such as P%. However, harvested tomato fruits of all treatments, except the self-grafted Peto 86, recorded high values of N and K% in both seasons.

### 2.5. Fruit Quality Parameters

**Figure 3:**
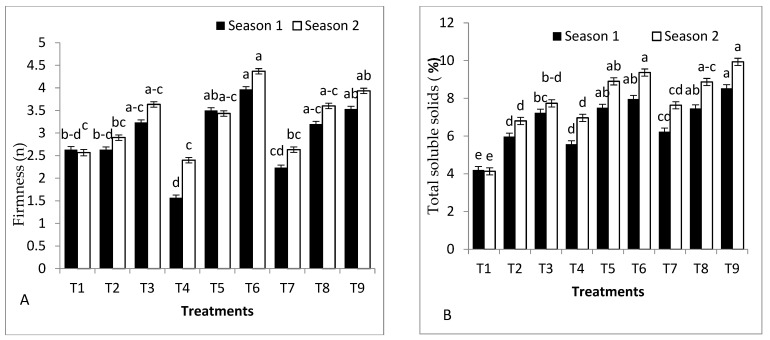

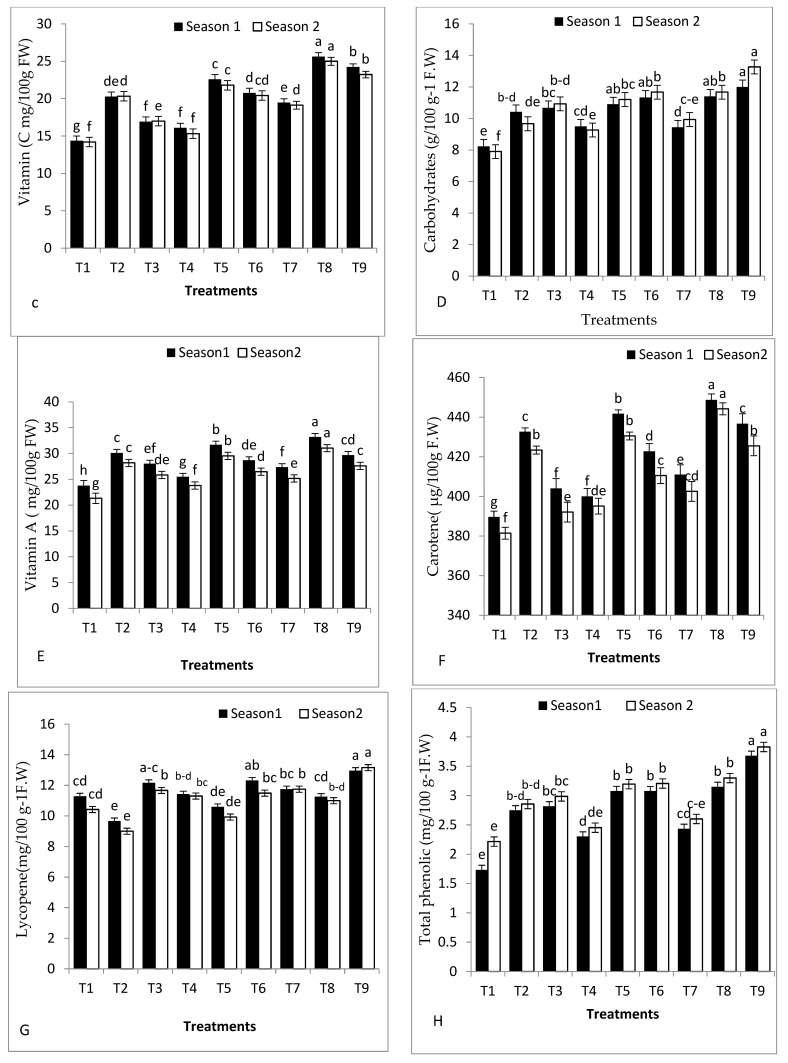
Effects of grafting, rootstock seed treated with SA, and colonisation with *S. griseus* on (**A**) firmness, (**B**) total soluble solids, (**C**) vitamin C, (**D**) total carbohydrates, (**E**) vitamin A, (**F**) Carotene, (**G**) lycopene, and (**H**) total phenolic in the first and second seasons. Treatments: T1—self-grafted Peto 86/Peto 86 (control); T2—Peto 86/*S. cheesmaniae*; T3—Peto 86/GS hybrid; T4—Peto 86/Peto 86 + SA; T5—Peto 86/*S. cheesmaniae* + SA; T6—Peto 86/GS hybrid + SA; T7—Peto 86/Peto 86 + *S. griseus; T8*—Peto 86/*S. cheesmaniae* + *S. griseus*; and T9—Peto 86/GS hybrid + *S. griseus*. Standard errors of the mean are shown as vertical bars; the LSD test indicates that differences between values in each bar that are marked by different letters are significant at *p* = 0.05.

**Figure 4:**
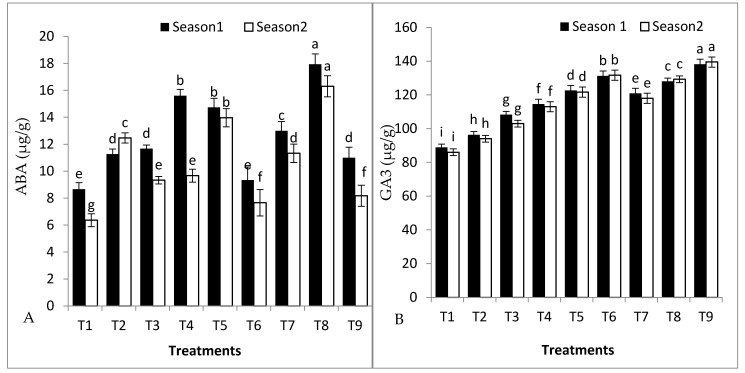

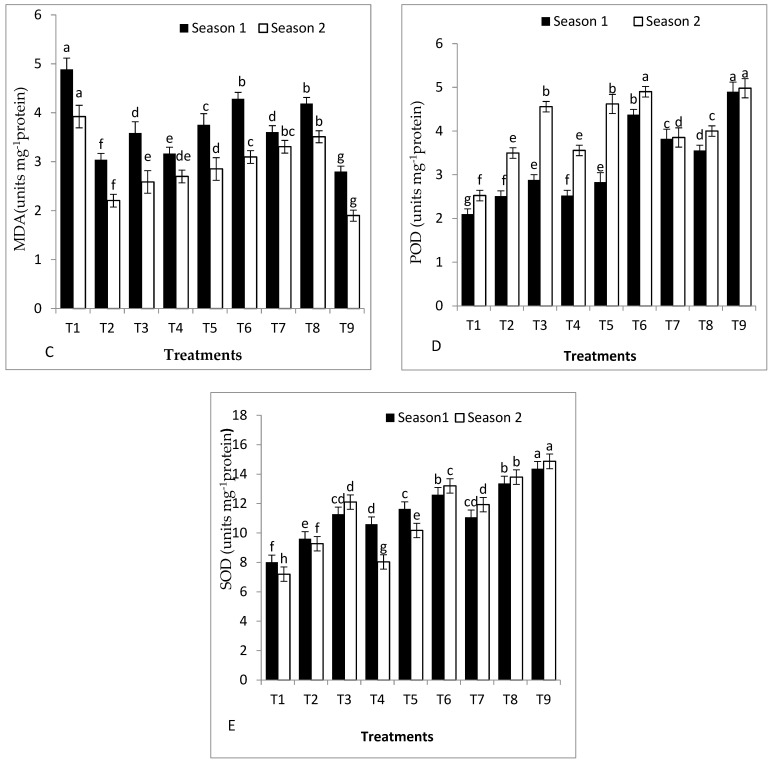
Effects of grafting, rootstock seed treated with SA, and colonisation with *S. griseus* on (**A**) GA3, (**B**) ABA, (**C**) MDA, (**D**) POD, and (**E**) SOD in the first and second seasons. Treatments: T1—self-grafted Peto 86/Peto 86 (control); T2—Peto 86/*S. cheesmaniae*; T3—Peto 86/GS hybrid; T4—Peto 86/Peto 86 + SA; T5—Peto 86/*S. cheesmaniae* + SA; T6—Peto 86/GS hybrid + SA; T7—Peto 86/Peto 86 + *S. griseus*; T8—Peto 86/*S. cheesmaniae* + *S. griseus;* and T9—Peto 86/GS hybrid + *S. griseus.* Standard errors of the mean are shown as vertical bars; the LSD test indicates that differences between values in each bar that are marked by different letters are significant at *p* = 0.05.

The quality parameters of tomato fruits were drastically influenced by grafting, rootstock seed treatments, and their combined interactions (Figure 3). Firmness displayed insignificant variation among self-grafted Peto 86 and other treatments in both seasons (Figure 3A). Harvested tomato fruits from all treatments, except the self-grafted Peto 86, recorded high values of total soluble solids in both seasons (Figure 3B). Total carbohydrates of tomato fruits were considerably influenced by grafting, rootstock seed treatments, and their combinations in both seasons (Figure 3C). Tomato fruits obtained from *S. cheesmaniae* rootstock with *S. griseus* increased the content of vitamin C by 78.35 and 76.05%, vitamin A by 39.54% and 45.47%, and carotene by 15.16% and 16.46% compared to the control treatment in the first and second seasons (Figure 4D–F). Nevertheless, harvested tomato fruits from *S. cheesmaniae* rootstock had the lowest content of lycopene compared to control plants in both seasons (Figure 3G). Tomato fruits obtained from GS hybrid with *S. griseus* induced an increase in the content of total phenols by 111.7% and 71.4% compared to control plants in the first and second seasons, respectively. On the contrary, the lowest content of total phenols was obtained from self-grafted Peto 86 in the first and second seasons (Figure 3H).

### 2.6. Antioxidant Enzymes, Plant Hormones, and Malondialdehyde Content in Tomato Leaves

The effects of grafting, rootstock seed priming, and their combined interactions on the activity of the most important hormones, gibberellic acid (GA3), abscisic acid (ABA), peroxidase (POD), and Superoxide dismutase (SOD) antioxidant enzymes, as well as malondialdehyde (MDA), are shown in (Figure 4A–E). The higher GA3 content was registered for GS hybrid combined with *S. griseus* in the first and second seasons. In contrast, the self-grafted Peto 86 exhibited the lowest concentration of GA3 in the first and second seasons. Moreover, GS hybrid combined with *S. griseus* and GS hybrid combined with SA exhibited better POD activity in both seasons. Moreover, GS hybrid combined with *S. griseus* recorded the highest SOD activity in the first and second seasons. Correspondingly, self-grafted Peto 86 had the lowest POD and SOD activity in both seasons. A higher MDA content was recorded for self-grafted Peto 86 in the first and second seasons. On the contrary, a lower MDA content was recorded for GS hybrid combined with *S. griseus* in the first and second seasons. Combined *S. cheesmaniae* rootstock with *S. griseus* registered the highest content of ABA in leaves in the first and second seasons. Despite this, self-grafted Peto 86 registered the lowest content of ABA in the first and second seasons.

### 2.7. Cluster Analysis

**Figure 5:**
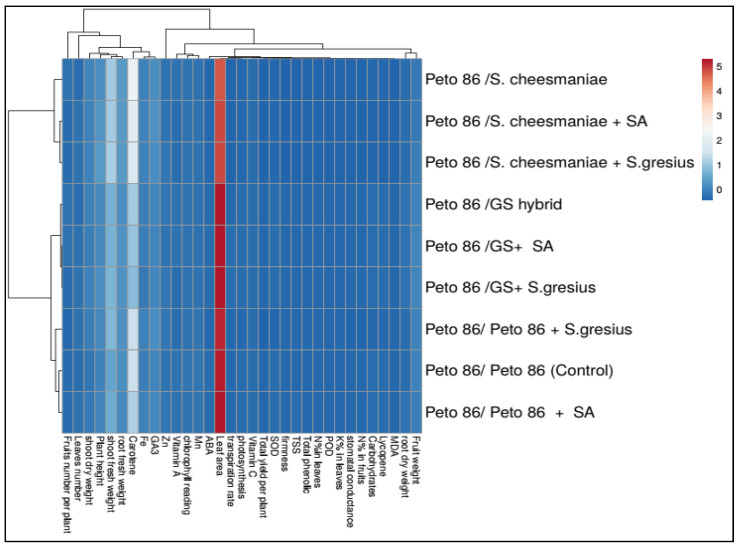
Heatmap of grafting, rootstock seeds treated with SA, and colonisation with *S. griseus*, and measured attributes of tomato plants. The heatmap diagram shows the variations in the response variables across all investigated treatments. Individual response variables are represented by columns, while treatments are represented by rows. According to the scale in the bottom right corner of the heatmap, lower numerical values are coloured blue and higher numerical values are coloured red.

Cluster analysis that included morphological and physiological traits, in addition to biochemical ingredients, are shown in Figure 5. The overall variances among all treatments were easily revealed by heatmap analysis. The cross-grafting treatment alone increased productivity compared to self-grafted Peto 86 (control). Additionally, rootstock seeds colonised with *S. griseus* of had a beneficial effect through the mitigation of oxidative injury by improving photosynthetic performance, raising antioxidant enzyme concentrations, and drastically promoting plant growth and production under cold stress.

### 2.8. Trait Interrelationships

**Figure 6:**
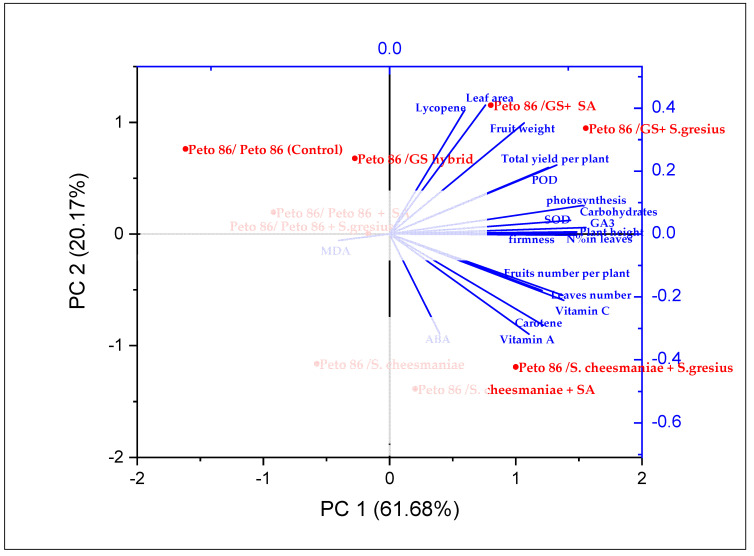
Biplot of the principal components for plant growth, total yield, and physiochemical attributes of tomato plants. The plant growth parameters comprise leaf number per plant, plant height, and leaf area. The yield parameters include number of fruits per plant, fruit weight and total yield per plant. The fruit quality attributes include firmness, vitamin C, vitamin A, lycopene content, carotene, total carbohydrates, and total phenol. The physiological parameters include photosynthesis and stomatal conductance. The physiochemical parameters comprise SOD, POD, GA3, MDA, and ABA. Red circle symbols signify the nine treatments: self-grafted Peto 86/Peto 86 (control), Peto 86/*S. cheesmaniae*, Peto 86/GS hybrid, Peto 86/Peto 86 + SA, Peto 86/*S. cheesmaniae* + *SA, Peto 86/GS* hybrid + SA, Peto 86/Peto 86 + *S. griseus*, Peto 86/*S. cheesmaniae* + *S. griseus*, and Peto 86/GS hybrid + *S. griseus*.

The association among the evaluated physiological, biochemical, yield, and growth characteristics of tomato plants was measured based on the analysis of principal components. Figure 6 displays the score plots of different treatments and control plants. The sum of the first and second principal components (PC1 and PC2) accounted for 81.85% of variation between tomato plants. PC1, the first component, contributed 61.68% of the overall variation, and the second component (PC2) accounted for 20.17% of the overall variation. GS hybrid combined with *S. griseus* or GS hybrid combined with SA exhibited improved plant growth with higher plant height, leaf area, fruit weight, and total yield. In addition, they showed increased photosynthesis, nitrogen content, antioxidant enzymes (SOD and POD), and GA3 in leaves. In addition, harvested tomato fruit GS hybrid combined with *S. griseus* improved total carbohydrates and lycopene. *S. cheesmaniae* rootstock with *S. griseus* or *S. cheesmaniae* rootstock with SA exhibited a higher leaf number and fruit number per plant. They also exhibited higher quality, including vitamin C, vitamin A, firmness, and carotene. In addition, they displayed higher concentrations of leaf ABA under cold stress conditions. The inter-relationship between the evaluated characteristics (Figure 6) indicates that the yield parameters are positively associated with plant height, leaf area, photosynthesis, fruit weight, antioxidant enzymes (SOD and POD), and GA3. However, the high values of the photosynthesis rate are associated with the greater total yield and its contributing traits. Furthermore, the MDA content exhibited a highly negative association with photosynthesis under cold stress conditions.

### 2.9. Leaf Anatomy

**Figure 7:**
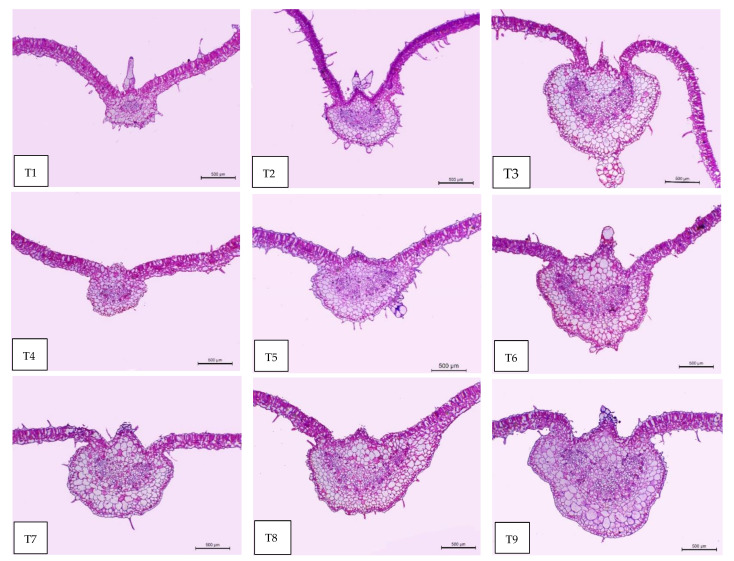
Microphotographs of cross sections through the blade of the terminal leaflet of the fourth compound leaf developed on the main stem of tomato plants, aged 60 days. Scale bars = 500 µm. T1—self-grafted Peto 86 as a control; T2—Peto 86/*S. cheesmaniae;* T3—Peto 86/GS hybrid; T4—Peto 86/Peto 86 + SA; T5—Peto 86/*S. cheesmaniae* + SA; T6—Peto 86/GS hybrid + SA; T7—Peto 86/Peto 86 + *S. griseus*; T8—Peto 86/*S. cheesmaniae* + *S. griseus*; T9—Peto 86/GS hybrid + *S. griseus*.

**Figure 8:**
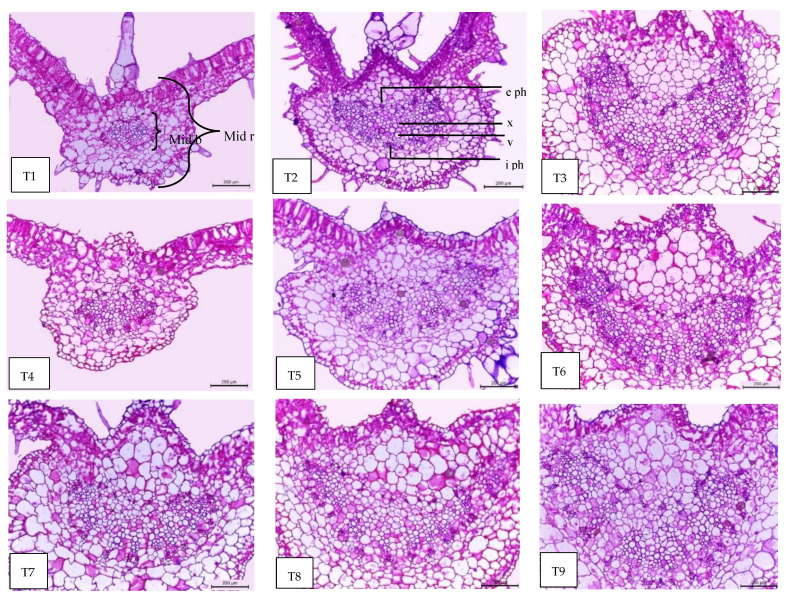
Magnified portions of the leaf blade midvein of tomato plants, aged 60 days. Scale bars = 200 µm. T1—self-grafted Peto 86 as a control; T2—Peto 86/*S. cheesmaniae*; T3—Peto 86/GS hybrid; T4—Peto 86/Peto 86 + SA; T5—Peto 86/*S. cheesmaniae* + SA; T6—Peto 86/GS hybrid + SA; T7—Peto 86/Peto 86 + *S. griseus*; T8—Peto 86/*S. cheesmaniae* + *S. griseus*; T9—Peto 86/GS hybrid + *S. griseus*. Details: mid b, midvein bundle; mid r, midvein region; e ph, external phloem; i ph, internal phloem; v, vessel; x, xylem.

**Figure 9:**
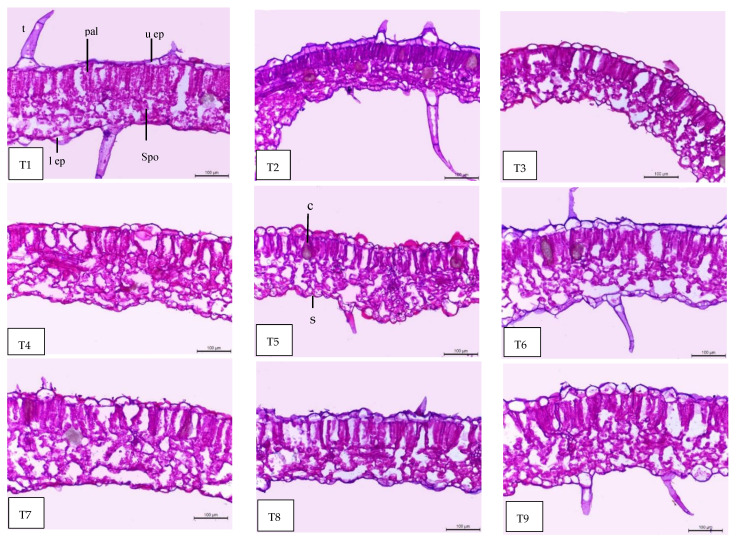
Magnified portions of the leaf blade lamina of tomato plants, aged 60 days. Scale bars = 100 µm. T1—self-grafted Peto 86 as a control; T2—Peto 86/*S. cheesmaniae*; T3—Peto 86/GS hybrid; T4—Peto 86/Peto 86 + SA; T5—Peto 86/*S. cheesmaniae* + SA; T6—Peto 86/GS hybrid + SA; T7—Peto 86/Peto 86 + *S. griseus*; T8—Peto 86/*S. cheesmaniae* + *S. griseus*; T9—Peto 86/GS hybrid + *S. griseus*. Details: l ep, lower epidermis; spo, spongy tissue; pal, palisade tissue; u ep, upper epidermis; t, trichome; c, cells with dark content containing calcium oxalate crystals; s, stomata.

Regarding the anatomical features, the tomato leaf has an epidermis with glandular and non-glandular trichomes and stomata on both sides. The mesophyll consists of palisade cells facing the lamina upper epidermis and the spongy cells facing the lower epidermis. In the midrib, the vascular bundle of the midrib is bicollateral with phloem facing both sides of the leaf and is surrounded by ground parenchyma (Figure 7, Figure 8 and Figure 9). It can be observed in Table 5 and Figure 7, Figure 8 and Figure 9 that all treatments under low-temperature conditions achieved an improvement in all anatomical characteristics under study, compared to self-grafted Peto 86 (control). Peto 86/GS hybrid + *S. griseus* treatment resulted in the greatest increase in the thickness of both midvein and lamina by 76.61% and 30.41%, respectively, compared to the control. The promotive effect on the leaf lamina was achieved due to an increase in the thickness of palisade tissue, spongy tissues, upper epidermis, and lower epidermis by 14.98, 53.84, 18.59, and 19.57%, respectively, compared to the control. Likewise, the vascular bundle of the midvein also increased as a result of the increase in length, width, and mean diameter of vessels by 88.98, 128.56, and 120.39%, respectively, compared to the control (Figure 7, Figure 8 and Figure 9 T1 and T9). A minimum increase was achieved with the Peto 86/Peto 86 + SA treatment for the thickness of the midvein, the midvein bundle length and width, and the mean diameter of vessels by 14.65, 4.04, 24.56, and 5.79%, respectively, more than the control. In addition, the thickness of the upper epidermis and the thickness of the lower epidermis increased by 5.01 and 2.89%, respectively, more than the control (Figure 7 and Figure 8 T1 and T4). The treatment of Peto 86/*S. cheesmaniae* showed a decrease in the thickness of the lamina, the thickness of palisade tissue, and the thickness of spongy tissue by 39.07, 46.18, and 28.29%, respectively, less than the control (Figure 9 T1 and T2).

## 3. Discussion

During the period of April to May, Egypt’s vegetable markets generally suffer a major lack of tomato fruit crop availability. The grafting phenomena could lessen the seasonal reduction in tomato production. In the current assessment, the fruit harvest from the late winter planting began in March and continued through April and May. Plant growth parameters are a perfect indicator for evaluating several abiotic stresses on plants (Table 1 and Table 2). The results also illustrate that low temperature and grafting onto Peto 86 reduced plant height and the fresh and dry weight of shoots and roots. These results are in accordance with those obtained by the authors of [27]. Under cold stress, significant changes in plant growth parameters were enhanced in the scion Peto 86 when grafted onto GS hybrid and *S. cheesmaniae* tolerance rootstock. This obviously shows the role of the rootstock genotype on the transcriptional reactions of the scion even when the roots are subjected to cold stress [28]. Genotypic variations in the reactions of tomato shoot growth to low temperature have been studied [29]. Moreover, the present data indicate that grafting with colonised seeds of rootstock with *S. griseus* is an alternative method for enhancing the cold tolerance of tomato fruits. The plant growth of the most grafted combinations with *S. griseus* was significantly higher than their counterparts without *S. griseus* colonisation. The combination of grafting and *S. griseus* could enhance the cold stress of tomato plants. Streptomyces induces plant growth through biological nitrogen fixation and phosphorous and potassium solubilisation. Through these procedures, inaccessible sources of nitrogen, phosphorus, and potassium are transformed into accessible soft sources [30]. Due to specific climatic changes and environmental factors, several soil-dwelling micro-organisms that are connected to plants alter their behaviour [31].

The results also indicate that both low temperatures reduced the total yield and fruit number per plant (Figure 1A–D). The negative effect of cold stress on the total yield is due to slower CO_2_ fixation and fruit photosynthetic partitioning [32]. These results are in agreement with the findings in [33]. In particular, under low temperature, the use of GS hybrid as rootstock was associated with a significantly higher fruit weight and total yield compared to *S. cheesmaniae* rootstock. It should be noted that the rootstock genotype had a significant role in the performance of the grafted tomato under stress conditions [34]. The combination of grafting with SA application was associated with a significantly higher fruit number, fruit weight, and total yield per plant values under cold stress conditions. Our results are strongly supported by the findings in [22], where in researchers reported that SA enhanced plant growth and productivity of cowpea plants. The positive effects of SA acid on the total yield may be owed to the photo assimilation translocation to fruits, thereby enhancing fruit weight [23]. Moreover, the results demonstrate that the use of rootstocks inoculated with *S. griseus* (particularly GS hybrid) increased the total yield per plant by 10.5% and 5.7% in the first and second seasons, respectively. The appreciated influence of *S. griseus* on the total yield may be due to the creation of indole-3-acetic acid (IAA), which improves seed germination, root dry weight, and root elongation on tomato [35].

The results also illustrated that sub-optimal temperature and grafting on to Peto 86 reduced photosynthetic pigments in both seasons (Figure 2A–D). Cold stress negatively affects and reduces the plant’s water and nutrient availability, which results in a decreased photosynthetic rate [36]. The scion of Peto 86 exhibited higher nutrient availability and photosynthetic rate when grafted onto GS hybrid rootstock and *S. cheesmaniae* rootstock compared to self-grafted plants. There is a significant contribution made to growth by the shoot genotype [34]. Cross-grafting involves tissue wounding and reconnection, similar to what is seen in wound healing or self-grafting; hence, it may be assumed that certain compatibility variables may contribute to increased stress tolerance. These could involve specific root-to-shoot signals [37] and RNA transport [38]. In this study, the combination of grafting with SA application was associated with significantly higher chlorophyll content (SPAD), transpiration rate, photosynthesis, and stomatal conductance in both seasons. These results are strengthened by those found in [22,32]. The highest values of chlorophyll content, transpiration rate, photosynthesis, and stomatal conductance appeared when using GS hybrid inoculated with *S. griseus* in both seasons. Many studies have illustrated that isolates S*treptomyces niveus, Streptomyces rochei, Streptomyces pada, Streptomyces coelicolor, Streptomyces olivaceus,* and *Streptomyces geysiriensis* may play a vital role in mitigating drought and salinity stress in different crops by changing the physiological traits of plants [39] The appreciated effect of *S. griseus* on physiological properties may be due to the role of phytohormones, which play a crucial role in the regulation of plant cells and physiological processes.

According to the results (Figure 3A–H), tomato fruit quality traits, such as TSS, total carbohydrates, vitamin C, vitamin A, lycopene, carotene, and total phenol, are changed due to cold stress. No significant differences among treatments were observed in firmness in both seasons. However, harvested tomato fruits from all treatments recorded high values of total carbohydrates in both seasons compared to the control (Figure 3C). These results are consistent with those found in [40]. Lycopene content is a crucial factor to be considered when evaluating fruit colour because it is the main pigment responsible for the colour of mature tomatoes [41]. There is a significant increase in lycopene content and total phenols in tomato fruits obtained from GS hybrid with *S. griseus*, and tomato fruits obtained from GS hybrid with shikimic acid in both seasons. Similar data were obtained by the authors of [22], who used SA to enhance fruit quality. Tomato fruits obtained from *S. cheesmaniae* rootstock with *S. griseus* showed significantly greater vitamin C, vitamin A, and carotene compared to control plants. Similar data were obtained for tomato *S. cheesmaniae* rootstock by the authors of [41], who studied the genetic variability and performance of the tomato *S. cheesmaniae* genotype for yield and fruit quality. The authors of [42] reported that bio-priming of pumpkin seeds treated with a combination of *Azospirillum, phosphobacteria,* and *Pseudomonas fluorescens* improved plant growth, seed yield, and quality.

The self-grafted Peto 86 showed the lowest concentrations of nutrients including N, P, K, Ca, Mn, Fe, and Zn under cold stress conditions (Table 3). On the contrary, the data for *S. griseus* combined with grafting showed increased absorption of minerals under cold stress conditions. Combined grafting with SA application significantly realised higher leaf mineral content in both seasons. These results are strengthened by those reported in [22].

Tomato fruits harvested from GS hybrid with *S. griseus* recorded the highest content of N and K% compared to control plants and other treatments in both seasons (Table 4). Many *Streptomyces* species that have been isolated from rhizosphere soils have been illustrated to be superior phosphate solubilisers and promote plant growth [43]. Streptomyces has the potential to release potassium from silicate minerals and has been found to degrade potassium-bearing rock powder by solid-state fermentation [44]. The majority of *Streptomyces* isolates have a nitrogen fixing gene [45] and strong nitrogen fixing abilities [46].

Phytohormones play a major part in plant growth, especially under cold stress. The results indicate that both sup-optimal temperature and grafting onto Peto 86 reduced GA3 concentration and ABA in both seasons (Figure 4A,B). High content of ABA was registered by *S. cheesmaniae* rootstock with *S. griseus* in both seasons. GA3 and ABA are responsible for regulating plant growth under cold stress [47]. Plants have created a variety of enzymatic defence mechanisms to detoxify free radicals and lessen oxidative stress in order to decrease the oxidative damage caused by abiotic stress, including cold stress. Cold stress reactions are typically correlated with changes in antioxidant enzyme activity [47]. The data recorded in Figure 4D,E clearly indicate that GS hybrid combined with SA significantly improved the activity of POD and SOD in tomato leaves during the exposure to the low temperature of the winter season. The highest mean values of POD and SOD activity were recorded in plants grafted onto GS hybrid colonised with *S. griseus* compared to the control and other treatments in both seasons. Similar results have also been confirmed by the authors of [48].

Malondialdehyde is one of many low-molecular-mass end products that result from the breakdown of many primary and secondary lipid peroxidation chemicals [49]. Therefore, increased ROS production as a result of oxidative stress is indicated by the increase in the MDA level in plant tissue. High MDA content was recorded for self-grafted Peto 86 (Figure 4C). On the contrary, low MDA content was recorded for GS hybrid combined with *S. griseus* in both seasons. The enzymes POD and SOD are just two of the many enzymatic defence mechanisms that plants might use to reduce the harmful effects of ROS [49].

Based on the anatomy of tomato leaves, low-temperature stress induces changes in growing plants, including leaf initiation, root cell division, and elongation. The reduction in root growth results in reduced water and nutrient uptake, leading to reduced nutrient use efficiency. Low-temperature stress causes dysfunction of the photosynthesis apparatus localised inside the chloroplast and decreases light absorption in the thylakoid electron transport of the photosystem, as well as causing a negative effect on the mechanics of opening and closing stomata. The stomatal closure has negative effects on the leaf gas exchange due to the limitation of CO_2_ supply [50].

The treatment with SA at a 60 ppm concentration showed an improvement in all anatomical characteristics under study (thickness of the midvein, lamina, palisade tissue, spongy tissue, upper and lower epidermis, bundle dimension (length and width), and mean vessels diameter). This effect is attributed to the fact that shikimic acid stimulates growth parameters, leaf area, transpiration rate, and photosynthetic pigments [22]. On the other hand, SA may exert its effect on photosynthetic machinery at the mesophyll and chloroplast level by increasing plastid biogenesis through its action on increasing the biosynthesis of indole acetic acid (IAA) from tryptophan. The strong enhancing effect of all anatomical features of grafted tomato plants when treated with *S. griseus* might be attributed to the direct effect of improving growth by the production of phytohormones, including auxins, gibberellins, cytokinins, siderophores scavenging ferric iron from the environment, nitrogen fixation, and the suppression of stress in plants by the production of 1-aminocyclopropane-1-carboxylate [51,52].

## 4. Materials and Methods

### 4.1. The Experimental Site, Plant Material, and the Tested Compounds 

**Figure 10:**
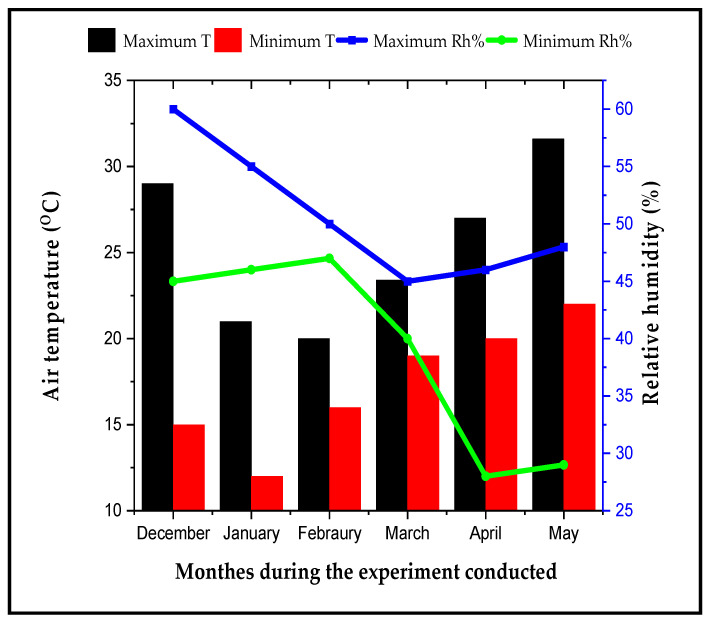
Monthly weather data of The Experimental Farm, Faculty of Agriculture, Cairo University, Giza, Egypt. Minimum air temperature (minimum T (°C)), maximum air temperature (maximum T(°C)), minimum relative humidity (Min Rh%), and maximum relative humidity (Max Rh%).

Two open-field experiments were performed in The Experimental Farm, Vegetable Crops Department, Faculty of Agriculture, Cairo University, Giza, Egypt (31°1′10′′ N latitude and 30°12′4′′ E longitude), during two consecutive winter seasons (December to April) of 2020 and 2021. The meteorological data of the experimental location are presented in Figure 10. The cultivar Peto 86, tomato (obtained from Moon Star Co., Pakistan) was employed as a scion. The seeds of tomato rootstock, wild tomato accession *S. cheesmaniae* L. (line LA 524), were obtained from Dr. Charles Block, Plant Introduction Station, Ames, Iowa. In addition, GS hybrid that was obtained from Mecca Trade Company, Cairo, Egypt was used as a commercial cultivar with recognised tolerance qualities. Shikimic acid was purchased from Technogene Company, Egypt. SA was used to prepare the SA solution by dissolving 10 mg of SA in 1 mL of distilled water. The product name is New-Acteno, which contains isolated *Streptomyces griseus (MT210913*) and was used at a dilution of 1:50 (1 mL contain 107 CFU). It was produced by the Central Lab of Organic Agriculture (CLOA), Agriculture Research Centre, Giza, Egypt.

### 4.2. Seed Treatments 

Before sowing, the seeds of tomato rootstocks Peto 86, *S. cheesmaniae*, and GS hybrid were sterilised in 7% sodium hypochlorite for 10 min and then washed well with distilled water. The sterilised seeds were split into three groups. The first one was named the control treatment. The second group was soaked in SA at a 60 ppm concentration. For priming with SA, rootstock seeds were immersed in a priming solution of shikimic acid at 20 °C for 4 h in the dark [53]. After that, the seeds were washed five times with distilled water for six minutes each and dried using blotting paper and a flow of dry air at 30 °C. The third group of sterilised seeds was soaked in 1 mL of *S. griseus* spore suspension (1 × 107 CFU/mL), incubated with bacterial suspensions at room temperature for 6 h, and then left to dry under the laminar flow hood [54]. 

### 4.3. Grafting and Growth Conditions 

On December 1st and 3rd of 2020 and 2021, scion seeds and all groups of rootstock seeds were planted in seedling trays filled with vermiculite and peat moss (1:1 v:v). On December 25th and 27th of 2020 and 2021, scion seedlings were grafted onto rootstock using the tongue grafting technique described by the authors of [55]. The successfully grafted tomato plants were transplanted in the open field on January 7th in 2021 and on January 10th in 2022. Grafted tomato plants were transplanted in the field 35 cm apart on the northern side of ridges measuring 1.5 m wide and 14 m long. The experimental design includes 9 treatment combinations as follows: (T1) self-grafted Peto 86/Peto 86 (control); (T2) Peto 86/*S. cheesmaniae*; (T3) Peto 86/GS hybrid; (T4) Peto 86/Peto 86 + SA; (T5) Peto 86/*S. cheesmaniae* + SA; (T6) Peto 86/GS hybrid + SA; (T7) Peto 86/Peto 86 + *S. griseus*; (T8) Peto 86/*S. cheesmaniae* + *S. griseus*; and (T9) Peto 86/GS hybrid + *S. griseus.* The treatments were arranged in a randomised complete block design (RCBD) with three replicates. All agricultural and farming practices for tomato crops in the open field were performed as recommended by the Egyptian Ministry of Agriculture [56]. The soil is clay loam in texture, EC 1.3 dS m^−1^, and contains 119 mgL^−1^ N, 24 mgL^−1^ P, and 31 mgL^−1^ K. The pH value is 7.04. The soluble cation values are 4.6, 3.3, 2.1, and 3.4 meq. L^−1^ for Ca^++^, Mg^++^, K^+^, and Na^+^, respectively, and 0.5, 4.1, and 7.2 meq. L^−1^ for HCO^3−^, Cl^−^, and SO^4^, respectively.

### 4.4. Data Recorded

#### 4.4.1. Plant Growth and Yield 

After 60 days from transplanting, 10 tomato plants were selected to measure plant height and average number of leaves and leaf area was measured by the laser area meter CI-202 USA. To determine the dry weights of shoots and roots, each of fresh sample was weighed and dried in an oven at 70 °C until reaching constant weight. During harvest, the fruits of each plot were collected, and the fruit fresh weight, fruit number per plant, and total fruit yield per plant were recorded.

#### 4.4.2. Gas Exchange and Chlorophyll Measurements of Tomato Plants

Gas exchange parameters (transpiration rate, net photosynthesis, and stomatal conductance) were measured using an infrared gas analyser (LICOR 6400 Portable Photosynthesis System; IRGA, Licor Inc., Lincoln, NE, USA). In terms of the photosynthetic parameters, the fourth leaf of each tomato plant were selected from each treatment at 60 days from transplanting to measure photosynthesis and all measurements were performed between 10:30 a.m. and 14:00 p.m. with a light intensity of 1200 mol m^−2^ s^−1^ and 85% RH. The temperature of the field ranged from 22.2 to 24.3 °C. The volume of gas flow rate was 390 mL min^−1^. The content of CO_2_ in the air was 397 µmol mol^−1^. The fourth leaf from each treatment was used to measure the amount of leaf chlorophyll using a SPAD meter (SPAD-502, Konica Minolta Sensing, Inc., Osaka, Japan) according the method described by [57].

#### 4.4.3. Fruit Quality

Ten mature tomato fruits per experimental plot were selected to measure the following properties:

Total soluble solids percentage (TSS %) was assessed by a Zeiss laboratory refractometer. Five fruits were collected in each replicate to measure their firmness. The firmness of each ripe tomato fruit was determined by a Force GaugeMode M4-200 (ELECTROMATIC Equipment Co., Inc., Cedarhurst, NY, USA) with a 1 mm diameter flat probe. The firmness readings of tomato fruits were made at two opposing points of the equatorial positions and expressed in newtons. 

Total carbohydrates in fruit were determined by the phosphomolybdic acid method according to [58], where 2 gm of sample was mashed with 10 mL 80% ethanol with mortar and pestle and then filtered using Whatman filter paper. The filter and residue were gathered separately. The alcohol residue was added to a 250 mL conical flask along with 150 mL distilled water and 5 ml concentrated HCL. The mixture was hydrolysed for 30 min and cooled to room temperature. Na_2_CO_3_ was added slowly until the extract became neutral (pH = 7). Then, this extract was filtered and the residue was discarded. The filtrate’s total volume was calculated. It served as a sample for total sugar. Each test tube included 0.5 mL of an aliquot sample and 1 mL of Somogyi’s reagent. For 30 min, all test tubes were immersed in boiling water, cooled to room temperature, and then given 1 mL of the arsenomolybdate reagent. The filtrate’s total volume was calculated. The content was mixed. Then, the content was diluted to a volume of 10 mL and its absorbance was measured at 560 nm by spectrophotometer.

Vitamin C as ascorbic acid (mg\100 g) was determined in fruits according to [58] method. Vitamin A (mg\100 g) was determined in fruits according to [59] method. Absorption determination for lycopene content was made by using Spectrophotometer UV-VISSPECORD 205 by Analytic Jena. Total carotenoid content was measured by spectrophotometer and calculated according to the equation described by [60]. Lycopene in the fruits of tomato samples was extracted with a hexane: ethanol: acetone (2:1:1) (*v*/*v*) mixture according to the method of [61]. Total phenolic content of the leaves was measured spectrophotometrically using the Folin–Ciocalteu colorimetric method [61]

#### 4.4.4. Mineral Content in Tomato Leaves and Fruits

Tomato samples were dried in an electric oven at 70 °C for 24 h according to [58] and then finely ground for chemical determination of elements. The wet digestion of 0.2 g plant material with sulphuric and perchloric acids was carried out on herbs by adding concentrated sulphuric acid (5 ml) to the samples and the mixture was heated for 10 min. Then, 0.5 mL perchloric acid was added and heating continued until a clear solution was obtained by [58]. The total nitrogen concentrations of the dried leaves and fruits was determined by using the modified micro-Kjeldahl method as described by [58]. Phosphorus concentrations of the dried leaves and fruits was determined calorimetrically by using the chlorostannousmolybdo phosphoric blue colour method in sulphuric acid according to the authors of [62]. In addition, potassium concentration was determined by using the flame photometer apparatus (CORNING M 410, Germany). Concentrations of micronutrients (Mn, Fe, and Zn) in leaf samples were determined using an atomic absorption spectrophotometer with air-acetylene and fuel (PyeUnicam, model SP-1900, US) [63].

#### 4.4.5. Antioxidants Enzymes, Hormones, and Malondialdehyde (MDA)

The leaf samples used to measure the activity of peroxidase (POD) were prepared according to [64], where (0.5 g) leaf tissue was frozen in liquid nitrogen to extract the peroxidase enzyme. The samples were ground with 10 mL extraction buffer (50 mM phosphate buffer, pH 7) containing 0.5 mM EDTA and 2% PVPP (*w*/*v*) and were centrifuged at 3930 rpm for 20 min. The resultant supernatant was used to determine the peroxidase activity using a spectrophotometric method by the formation of guaiacol in a l mL reaction mixture (450 µL 25 mMguaiacol, 450 µL 225 mM H_2_O_2_) and 100 µL crude enzymes. Superoxide dismutase (SOD) activity was assessed by assessing the inhibition of the photochemical reduction in the amount of nitro blue tetrazolium (NBT) [65,66]. One unit of superoxide dismutase (SOD) activity was defined as the amount of enzyme required for the reduction of 50% NBT.

Regarding the malondialdehyde (MDA) reaction, it was based on the methods explained by [67]. For HPLC analysis of MDA, all analyses were performed on a Waters Alliance 2690 HPLC system with an autosampler thermostatted at 8 °C and a column thermostatted at 40 °C. Detection was performed by a Waters 996 photodiode array detector. The system was controlled, and data were collected and integrated using Millennium Chromatography Manager software (version 4.0). Samples were analysed on a Rocket HPLC column, custom-packed with LiChrosorb RP C18 end-capped 3-m spherical particle size stationary phase (Alltech, Eke, Belgium). The column was protected with a guard cartridge packed with 5 m particles of the same material. The identity of the MDA peak was confirmed by coelution with authentic standards and by its characteristic absorption spectrum. The contents of gibberellic acid (GA3) and abscisic acid (ABA) in tomato leaves were analysed using the method described by [68]. A total of 15 mL of a mix containing methanol (80% *v*/*v*) and butylated hydroxytoluene was mixed with homogenised freeze-dried materials. More details on GA3 and ABA extraction and quantification can be obtained elsewhere AOAC [69].

### 4.5. Anatomical Studies

Tested specimens included the blade of the terminal leaflet of the fourth compound leaf developed on the main stem of tomato plants under low-temperature conditions, were taken throughout the second growing season of 2021/2022, 60 days after sowing. Approximately 1.0 cm of the specimens were killed and fixed in FAA solution (5 mL glacial acetic, 10 mL formalin, 35 mL water, and 50 mL ethyl alcohol 70%) for at least 48 h. The selected materials were washed in 30% ethyl alcohol, dehydrated in a normal ethanol and butyl alcohol series, embedded in paraffin wax with a melting point of 56 °C, sectioned to a thickness of 15 μm stained with crystal violet erythrosin, cleared in xylene, and mounted in Canada balsam in accordance with [70]. Transverse sections were completed with a Leica Microtome RM 2125 and then micrographed and measured using a Leica Light Image Analysis System DM 750 at the Faculty of Agriculture, Cairo University-Research Park (CURP). The following parameters were recorded: thickness of the midvein (μm), lamina (μm), palisade tissue (μm), spongy tissue (μm), upper and lower epidermis, bundle dimension (length and width) (μm), and mean vessels diameter (μm).

### 4.6. Statistical Analysis

Data of two growing seasons were statistically analysed by MSTATC software [71]. Analysis of variance (ANOVA) was carried out using LSD at *p* < 0.05 to test the significance between treatments [72]. In addition, heatmaps and principal component analysis (PCA) were completed using origin pro2021version software.

## 5. Conclusions

Based on our results, high tomato production under cold stress can be achieved by grafting their scions onto rootstock genotypes with cold tolerance. However, an outstanding tomato yield would be achieved from root-shoot exchange between cold-adapted genotypes. It can be concluded that grafting combined with colonisation of *S. griseus* is a unique method for enhancing cold tolerance and decreasing cold damage in tomato plants. Plant growth, total yield, fruit quality, mineral contents, GA3, ABA, SOD, and POD of grafted tomato combined with *S. griseus* were significantly higher than those of self-grafted Peto 86 under cold stress conditions. It is advisable to grow the cross-grafted Peto 86/GS hybrid combined with *S. griseus* or cross-grafted Peto 86/GS hybrid with SA at a 60 ppm concentration when tomato is cultivated under cold stress conditions.

## Figures and Tables

**Table 1:** Effects of grafting, rootstock seed treated with shikimic acid (SA), and colonisation with *Streptomyces griseus (SG)* on tomato plants growth during 2020 and 2021 seasons.

Treatment	Plant Height (cm)	Leaf Number	Leaf Area (cm^2^)
2020 season			
Peto 86/Peto 86 (Control)	71.0 ^i^	14.0 ^f^	1202 ^f^
Peto 86/*S. cheesmaniae*	104.0 ^g^	23.3 ^d^	855 ^i^
Peto 86/GS hybrid	132.7 ^e^	19.7 ^e^	1618 ^c^
Peto 86/Peto 86 + SA	98.33 ^h^	20.67 ^e^	1441 ^d^
Peto 86/*S. cheesmaniae* + SA	138.0 ^d^	35.33 ^b^	998.3 ^h^
Peto 86/GS + SA	157.7 ^c^	30.67 ^c^	1845 ^b^
Peto 86/Peto 86 + *SG*	115.0 ^f^	29.33 ^c^	1260 ^e^
Peto 86/*S. cheesmaniae* + *SG*	166.7 ^b^	43.67 ^a^	1102 ^g^
Peto 86/GS + *SG*	188.0 ^a^	36.33 ^b^	2037 ^a^
LSD 0.05	5.247	2.66	54.59
2021 Season			
Peto 86/Peto 86 (Control)	80.67 ^i^	17.67 ^g^	1208 ^d^
Peto 86/*S. cheesmaniae*	102.8 ^g^	26.00 ^e^	861.0 ^g^
Peto 86/GS	116.1 ^f^	23.00 ^f^	1624 ^c^
Peto 86/Peto 86 + SA	91.40 ^h^	23.67 ^f^	1258 ^d^
Peto 86/*S. cheesmaniae* + SA	131.7 ^d^	38.00 ^b^	1009 ^f^
Peto 86/GS + SA	148.7 ^c^	33.00 ^c^	1865 ^b^
Peto 86/Peto 86 + *SG*	123.4 ^e^	30.67 ^d^	1255 ^d^
Peto 86/*S. cheesmaniae* + *SG*	171.7 ^b^	47.00 ^a^	1109 ^e^
Peto 86/GS + *SG*	189.1 ^a^	39.83 ^b^	2049 ^a^
LSD 0.05	3.423	1.855	52.05

Values followed by different letters are significant according to LSD test (*p* ≤ 0.05%).

**Table 2:** Effects of grafting, seed priming with shikimic acid (SA), and colonisation with *S. griseus (SG)* on tomato plants growth during 2020 and 2021 seasons.

Treatments	Shoot Fresh Weight (g)	Shoot Dry Weight (g)	Root Fresh Weight (g)	Root Dry Weight (g)
2020 Season				
Peto 86/Peto 86 (Control)	183.3 ^g^	41.0 ^g^	115.2 ^h^	14.67 ^g^
Peto 86/*S. cheesmaniae*	252.0 ^f^	76. 7 ^e^	131.4 ^f^	21.0 ^f^
Peto 86/GS	297.3 ^d^	95.0 ^c^	142.1 ^e^	26.0 ^e^
Peto 86/Peto 86 + SA	242.7 ^f^	54.3 ^f^	122.3 ^g^	21.0 ^f^
Peto 86/*S. cheesmaniae* + SA	283.3 ^de^	85.0 ^d^	150.4 ^d^	29.0 ^d^
Peto 86/GS + SA	340.7 ^b^	101.3 ^b^	185.3 ^b^	38.0 ^b^
Peto 86/Peto 86 + *SG*	269.7 ^e^	79.3 ^e^	144.6 ^e^	24.7 ^e^
Peto 86/*S. cheesmaniae* + *SG*	322.3 ^c^	95.0 ^c^	162.7 ^c^	35. 3 ^c^
Peto 86/GS + *SG*	383.0 ^a^	114.3 ^a^	202.9 ^a^	44.7 ^a^
LSD 0.05	15.0	4.5	3.39	2.6
2021 Season				
Peto 86/Peto 86 (Control)	189.0 ^i^	39.33 ^i^	103.3 ^i^	13.00 ^h^
Peto 86/*S. cheesmaniae*	259.0 ^f^	81.00 ^g^	145.0 ^g^	21.00 ^f^
Peto 86/GS	306.2 ^d^	102.7 ^e^	166.7 ^e^	26.00 ^e^
Peto 86/Peto 86 + SA	224.9 ^h^	57.67 ^h^	121.7 ^h^	15.00 ^g^
Peto 86/*S. cheesmaniae* + SA	278.0 ^e^	97.00 ^f^	161.0 ^f^	28.67 ^d^
Peto 86/GS + SA	318.0 ^c^	118.7 ^c^	182.7 ^c^	34.0 ^c^
Peto 86/Peto 86 + *SG*	242.3 ^g^	115.3 ^d^	179.3 ^d^	25.00 ^e^
Peto 86/*S. cheesmaniae* + *SG*	361.2 ^b^	137.7 ^b^	201.7 ^b^	41.00 ^b^
Peto 86/GS + *SG*	391.9 ^a^	157.7 ^a^	221.7 ^a^	49.00 ^a^
LSD 0.05	8.867	2.8	2.804	1.942

Values followed by different letters are significant according to LSD test (*p* ≤ 0.05%).

**Table 3:** Effects of grafting, seed priming with shikimic acid (SA), and colonisation with *S. griseus (SG)* on nutrient content of tomato leaves during 2020 and 2021 seasons.

Treatment	Macronutrients (%) Micronutrients (ppm)						
	N%	P%	K%	Ca%	Mn ppm	Fe ppm	Zn ppm
2020 Season							
Peto 86/Peto 86 (Control)	2.2 ^f^	0.20 ^g^	2.10 ^e^	0.72 ^g^	35.3 ^g^	56.39 ^h^	29.29 ^g^
Peto 86/*S. cheesmaniae*	3.0 ^e^	0.3 ^ef^	3.31 ^d^	0.88 ^f^	37.10 ^f^	67.6 ^g^	37.00 ^f^
Peto 86/GS	3.4 ^d^	0.39 ^cd^	3.97 ^c^	1.09 ^d^	39.0 ^e^	79.3 ^f^	44.53 ^d^
Peto 86/Peto 86 + SA	2.8 e	0.28 ^f^	3.37 ^d^	0.85 ^f^	38.50 ^e^	80.9 ^e^	35.77 ^f^
Peto 86/*S. cheesmaniae* + SA	3.6 ^cd^	0.38 ^cd^	4.01 ^c^	1.12 ^d^	41.57 ^c^	85.0 ^d^	42.77 ^e^
Peto 86/GS + SA	4.3 ^b^	0.42 ^c^	3.82 ^c^	0.98 ^e^	40.27 ^d^	86.3 ^d^	42.97 ^e^
Peto 86/Peto 86 + *SG*	3.8 ^c^	0.36 ^de^	4.43 ^b^	1.22 ^c^	43.37 ^b^	88.0 ^c^	48.87 ^c^
Peto 86/S. c*heesmaniae* + *SG*	4.4 ^b^	0.53 ^b^	4.86 ^a^	1.29 ^b^	42.63 ^b^	90.5 ^b^	50.93 ^b^
Peto 86/GS + *SG*	4.9 ^a^	0.94	4.79 ^a^	1.46 ^a^	45.23 ^a^	92.5 ^a^	56.23 ^a^
LSD 0.05	0.26	0.05	0.30	0.07	0.96	1.61	1.279
2021 Season							
Peto 86/Peto 86 (Control)	1.19^i^	0.19 ^h^	1.43 ^f^	0.42 ^e^	33.13 ^i^	54.10 ^i^	28.15 ^h^
Peto 86/*S. cheesmaniae*	2.46 ^g^	0.27 ^g^	2.71 ^d^	0.57 ^e^	36.10 ^g^	58.23 ^g^	35.46 ^F^
Peto 86/GS	3.03 ^f^	0.3 ^fg^	3.57 ^b^	0.69 ^d^	37.20 ^f^	60.07 ^f^	40.35 ^e^
Peto 86/Peto 86 + SA	2.05 ^h^	0.36 ^f^	1.86 ^e^	0.55 ^e^	34.30 ^h^	55.53 ^h^	30.60 ^g^
Peto 86/*S. cheesmaniae* + SA	3.50 ^e^	0.44 ^e^	3.06 ^c^	0.78 ^d^	38.23 ^e^	62.0 ^e^	41.23 ^e^
Peto 86/GS + SA	4.09 ^d^	0.58 ^d^	2.70 ^d^	1.143 ^c^	39.13 ^d^	65.67 ^d^	44.06 ^d^
Peto 86/Peto 86 + *SG*	4.43 ^c^	0.64 ^c^	3.82 ^b^	1.25 ^b^	40.27 ^c^	81.67 ^c^	46.43 ^c^
Peto 86/*S. cheesmaniae* + *SG*	5.03 ^b^	0.73 ^b^	4.56 ^a^	1.317 ^ab^	41.30 ^b^	86.83 ^b^	48.51 ^b^
Peto 86/GS + *SG*	5.49 ^a^	0.91 ^a^	4.25 ^a^	1.36 ^a^	43.23 ^a^	89.5 ^a^	54.61 ^a^
LSD 0.05	0.077	0.054	0.32	0.09	0.78	1.21	1.254

Values followed by different letters are significant according to LSD test (*p* ≤ 0.05%).

**Table 4:** Effects of grafting, seed priming with shikimic acid (SA), and colonisation with *S. griseus (SG)* on macronutrients of tomato fruits during 2020 and 2021 seasons.

Treatment	N%	P%	K%
2020 Season			
Peto 86/Peto 86 (Control)	0.88 ^e^	0.005 ^a^	0.23 ^d^
Peto 86/*S. cheesmaniae*	1.16 ^d^	0.017 ^a^	0.31 ^bc^
Peto 86/GS	1.25 ^cd^	0.12 ^a^	0.35 ^b^
Peto 86/Peto 86 + SA	1.3 ^c^	0.019 ^a^	0.30 ^b–d^
Peto 86/S. cheesmaniae + SA	1.32 ^bc^	0.031 ^a^	0.33 ^b^
Peto 86/GS + SA	1.35 ^bc^	0.096 ^a^	0.37 ^b^
Peto 86/Peto 86 + *SG*	1.313 ^c^	0.031 ^a^	0.24 ^cd^
Peto 86/*S. cheesmaniae* + *SG*	1.430 ^ab^	0.12 ^a^	0.36 ^b^
Peto 86/GS + *SG*	1.473 ^a^	0.15 ^a^	0.5 ^a^
LSD 0.05	0.10	0.18 ns	0.07
2021 Season			
Peto 86/Peto 86 (Control)	0.52 ^c^	0.2 ^ab^	0.15 ^d^
Peto 86/*S. cheesmaniae*	1.12 ^b^	0.23 ^a^	0.23 ^bc^
Peto 86/GS	1.25 ^b^	0.26 ^a^	0.273 ^b^
Peto 86/Peto 86 + SA	1.20 ^b^	0.22 ^ab^	0.22 ^b–d^
Peto 86/*S. cheesmaniae* + SA	1.194 ^b^	0.25 ^a^	0.25 ^b^
Peto 86/GS + SA	1.137 ^b^	0.28 ^a^	0.29 ^b^
Peto 86/Peto 86+ *SG*	1.087 ^b^	0.26 ^a^	0.16 ^cd^
Peto 86/*S. cheesmaniae* + *SG*	1.243 ^b^	0.27 ^a^	0.27 ^b^
Peto 86/GS + *SG*	1.820 ^a^	0.28 ^a^	0.42 ^a^
LSD 0.05	0.44	0.077 ns	0.07

Values followed by different letters are significant according to LSD test (*p* ≤ 0.05%).

**Table 5:** Counts and measurements in micrometres (μm) of certain histological characteristics in transverse sections through the blade of the terminal leaflet of the fourth compound leaf developed on the main stem of tomato plants, aged 60 days, affected by grafting, rootstock seed treatments with shikimic acid (SA), and colonisation with *Streptomyces griseus (SG)* (means of three sections from three specimens).

Treatments	Histological Aspects								
	Thickness of Midvein	Thickness of Lamina	Thickness of Palisade Tissue	Thickness of Spongy Tissue	Dimensions of Midvein Bundle		Mean Diameter of Vessels	Thickness of Upper Epidermis	Thickness of Lower Epidermis
					Depth (Length)	Width			
Peto 86/Peto 86	628.68	158.86	95.74	69.11	168.12	305.68	13.63	19.14	14.51
Peto 86/*S. cheesmaniae*	755.42	96.79	51.52	45.25	260.17	414.47	14.96	20.80	15.18
Peto 86/GS hybrid	904.99	134.27	62.45	70.82	288.96	602.64	15.90	20.96	15.76
Peto 86/Peto 86 + SA	720.84	182.12	90.24	91.88	174.92	380.40	14.42	20.10	14.93
Peto 86/*S. cheesmaniae* + SA	790.27	160.72	64.39	96.33	267.20	537.21	15.09	21.83	15.26
Peto 86/GS hybrid + SA	934.32	135.87	64.25	72.45	315.41	650.28	16.05	21.33	16.98
Peto 86/Peto 86 + *S. griseus*	839.39	191.75	100.80	90.77	237.60	632.05	14.90	21.29	15.29
Peto 86/*S. cheesmaniae* + *S. griseus*	890.65	178.36	94.61	83.75	310.73	649.81	15.24	22.04	15.84
Peto 86/GS hybrid + *S. griseus*	1110.33	207.18	110.19	97.09	317.72	698.68	16.41	22.70	17.35

## Data Availability

Data is contained within the article.

## References

[1] FAOSTAT Crops and Livestock Products in 2020. http://www.fao.org/faostat/en/#data/QCL.

[2] Venema J.H., Posthumus F., Van Hasselt P.R. (1999). Impact of sub-optimal temperature on growth, photosynthesis, leaf pigments and carbohydrates of domestic and high-altitude wild Lycopersicon species. J. Plant Physiol..

[3] Schwarz D., Rouphael Y., Colla G., Venema J.H. (2010). Grafting as a tool to improve tolerance of vegetables to abiotic stress. Thermal stress, water stress and organic pollutants. Sci. Hortic..

[4] Abbas S.M. (2012). Effects of low temperature and selenium application on growth and the physiological changes in sorghum seedlings. J. Stress Physiol. Biochem..

[5] Mahajan S., Tuteja N. (2005). Cold, salinity and drought stresses: An overview. Arch. Biochem. Biophys..

[6] Gill S.S., Tuteja N. (2010). Polyamines and abiotic stress tolerance in plants. Plant Signal. Behav..

[7] Theocharis A., Clement C., Barka E.A. (2012). Physiological and molecular changes in plants grown at low temperatures. Planta.

[8] Kawaide T. (1985). Utilization of rootstocks in cucurbits production in Japan. Jpn. Agric. Res. Q..

[9] Yarsi G., Altuntas O., Sivaci A., Dasgan H.Y. (2017). Effects of salinity stress on plant growth and mineral composition of grafted and un-grafted Galia C8 melon cultivar. Pak. J. Bot..

[10] Knapp S., Spooner D.M., Peralta I. (2009). Taxonomy of wild tomatoes and their relatives (*Solanum* sect. *Lycopersicoides*, sect. *Juglandifolia*, sect. *Lycopersicon*; *Solanaceae*). Syst. Bot..

[11] Bedinger P.A., Chetelat R.T., McClure B., Moyle L.C., Rose J.K., Stack S.M., van der Knaap E., Baek Y.S. (2011). Interspecific reproductive barriers in the tomato clade: Opportunities to decipher mechanisms of reproductive isolation. Sex. Plant Reprod..

[12] Jisha K.C., Vijayakumari K., Puthur J.T. (2013). Seed priming for abiotic stress tolerance: An overview. Acta Physiol. Plant..

[13] Paparella S., Araújo S.S., Rossi G., Wijayasinghe M., Carbonera D., Balestrazzi A. (2015). Seed priming: State of the art and new perspectives. Plant Cell Rep..

[14] Rakshit A., Sunita K., Pal S., Singh A., Singh H.B. (2015). Biopriming mediated nutrient use efficiency of crop species. Nutrient Use Efficiency: From Basics to Advances.

[15] Sukanya V., Patel R.M., Suthar K.P., Singh D. (2018). An overview: Mechanism involved in biopriming mediated plant growth promotion. Int. J. Pure Appl. Biosci..

[16] Bisen K., Keswani C., Mishra S., Saxena A., Rakshit A., Singh H.B., Rakshit A., Singh H.B., Sen A. (2015). Unrealized potential of seed biopriming for versatile agriculture. Nutrient Use Efficiency: From Basics to Advances.

[17] Valli S., Suvathi S.S., Aysha O., Nirmala P., Vinoth K.P., Reena A. (2012). Antimicrobial potential of actinomycetes species isolated from marine environment. Asian Pac. J. Trop. Biomed..

[18] Shivlata L., Satyanarayana T. (2015). Thermophilic and alkaliphilic Actinobacteria: Biology and potential applications. Front. Microbiol..

[19] de Jesus Sousa J.N., Olivares F.L. (2016). Plant growth promotion by Streptomycetes: Ecophysiology, mechanisms and applications. Chem. Biol. Technol. Agric..

[20] Shao H.B., Chu L.Y., Jaleel C.A., Zhao C.X. (2008). Water deficit stress-induced anatomical changes in higher plants. C. R. Biol..

[21] Yao L., Wu Z., Zheng Y., Kaleem I., Li C. (2010). Growth promotion and protection against salt stress by Pseudomonas putida Rs-198 on cotton. Eur. J. Soil Biol..

[22] Aldesuquy H.S., Ibrahim H.A. (2000). The role of shikimic acid in regulation of growth, transpiration, pigmentation, photosynthetic activity and productivity of Vigna sinensis plants. Phyton.

[23] Elwan M.W.M., El-Hamahmy M.A.M. (2009). Improved productivity and quality associated with salicylic acid application in greenhouse pepper. Sci. Hort..

[24] Favati F., Lovelli S., Galgano F., Miccolis V., Di Tommaso T., Candido V. (2009). Processing tomato quality as affected by irrigation scheduling. Sci. Hort..

[25] Logemann E., Parniske M., Hahlbrock K. (1995). Modes of expression and common structural features of the completephenylalanine ammonia lyase gene family in parsley. Proc. Natl. Acad. Sci. USA.

[26] Lawton K., Weymann K., Friedrich L., Venooij B., Uknes S., Ryals J. (1996). Systemic acquired resistance in Arabidopsis requires salicylic acid not ethylene. Mol. Plant Microbe Interact..

[27] Venema J.H., Dijk B.E., Bax J.M., Van Hasselt P.R., Elzenga J.T.M. (2008). Grafting tomato (*Solanum lycopersicum*) onto the rootstock of a highaltitude accession of *Solanum habrochaites* improves sub-optimal-temperature tolerance. Environ. Exp. Bot..

[28] Ntatsi G., Savvas D., Papasotiropoulos V., Katsileros A., Zrenner R.M., Hincha D.K., Zuther E., Schwarz D. (2017). Rootstock Sub-Optimal Temperature Tolerance Determines Transcriptomic Responses after Long-Term Root Cooling in Rootstocks and Scions of Grafted Tomato Plants. Front. Plant Sci..

[29] Paul E.M.M., Hardwick R.C., Parker P.F. (1984). Genotypic variation in the response to sub-optimal temperatures of growth in tomato (*Lycopersicon esculentum* Mill.). New Phytol..

[30] Hamdali H., Bouizgarne B., Hafidi M., Lebrihi A., Virolle M.J., Ouhdouch Y. (2008). Screening for rock phosphate solubilizing actinomycetes from Moroccan phosphate mines. Appl. Soil Ecol..

[31] Glick B.R. (2012). Plant growth-promoting bacteria: Mechanisms and applications. Scientifica.

[32] Meena Y., Khurana D., Kaur N., Singh K. (2018). Towards enhanced low temperature stress tolerance in tomato: An approach. J. Environ. Biol..

[33] Ntatsi G., Hans-Peter K., Dietmar S. (2014). Growth, Yield, and Metabolic Responses of Temperature-stressed Tomato to Grafting onto Rootstocks Differing in Cold Tolerance. J. Am. Soc. Hortic. Sci..

[34] Sayed E.G., Mahmoud A.W.M., El-Mogy M.M., Ali M.A.A., Fahmy M.A.M., Tawfic G.A. (2022). TheEffective Role of Nano-Silicon Application in Improving the Productivity and Quality of Grafted Tomato Grown under Salinity Stress. Horticulturae.

[35] El-Tarabily K.A. (2008). Promotion of tomato (*Lycopersicon esculentun* Mill.) plant growth by rhizosphere competent 1-amino cyclopropane-1-carboxylic acid deaminase–producing Streptomycete actinomycetes. Plant Soil.

[36] Hajihashemi S., Noedoost F., Geuns J.M., Djalovic I., Siddique K.H. (2018). Effect of Cold Stress on Photosynthetic Traits, Carbohydrates, Morphology, and Anatomy in Nine Cultivars of *Stevia rebaudiana*. Front. Plant Sci..

[37] Aloni B., Cohen R., Karni L., Aktas H., Edelstein M. (2010). Hormonal signaling in rootstock–scion interactions. Sci. Hortic..

[38] Harada T. (2010). Grafting and RNAtransport via phloem tissue in horticultural plants. Sci. Hortic..

[39] Srivastava P., Kumar R. (2015). Soil salinity: A serious environmental issue and plant growth-promoting bacteria as one of the tools for its alleviation. Saudi J. Biol. Sci..

[40] Shi J., Le Maguer M. (2000). Lycopene in tomatoes: Chemical and physical properties affected by food processing. Crit. Rev. Food Sci. Nutr..

[41] Hassan A.A., Abdel-Ati K.E.A., Ibrahim T.A.A. (2020). Genetic behavior of some fruit characters in crosses between tomato and some wild species and among wild species. Plant Arch..

[42] Sivakalai R., Krishnaven K. (2017). Effect of Bio-Priming on Seed Yield and Quality in Pumpkin cv. CO_2_. Int. J. Curr. Microbiol. Appl. Sci..

[43] Hamdali H., Virolle M.J., von Jan M., Sproer C., Klenk H.P., Ouhdouch Y. (2011). *Streptomyces youssoufiensis* sp. nov., isolated from a Moroccan phosphate mine. Int. J. Syst. Evol. Microbiol..

[44] Liu D.F., Lian B., Wang B. (2016). Solubilization of potassium containing minerals by high temperature resistant *Streptomyces* sp. isolated from earthworm’s gut. Acta Geochim..

[45] Dahal B., NandaKafle G., Perkins L., Brozel V.S. (2017). Diversity of free-living nitrogen fixing Streptomyces in soils of the Badlands of South Dakota. Microbiol. Res..

[46] Sellstedt A., Richau K.H. (2013). Aspects of nitrogen-fixing Actinobacteria, in particular free-living and symbiotic Frankia. FEMS Microbiol. Lett..

[47] Zijian X.u., Jiachun W., Wentian Z., Tao S., Xiaohui H. (2022). Abscisic acid alleviates harmful effect of saline–alkaline stress on tomato seedlings. Plant Physiol. Biochem..

[48] Nourredine Y., Naima A., Dalila H., Habib S., Karim S. (2015). Changes of peroxidase activities under cold stress in annuals populations of Medicago. Mol. Plant Breed..

[49] Demiral T., Türkan I. (2005). Comparative lipid peroxidation, antioxidant defense systems and proline content in roots of two rice cultivars differing in salt tolerance. Environ. Exp. Bot..

[50] Wu J., Nadeem M., Galagedara L., Thomas R., Cheema M. (2022). Effects of Chilling Stress on Morphological, Physiological, and Biochemical Attributes of Silage Corn Genotypes during Seedling Establishment. Plants.

[51] Olanrewaju O.S., Babalola O.O. (2019). Streptomyces: Implications and interactions in plant growth promotion. Appl. Microbiol. Biotechnol..

[52] Mahmoud A.W.M., Ayad A.A., Abdel-Aziz H.S.M., Williams L.L., El-Shazoly R.M., Abdel-Wahab A., Abdeldaym E.A. (2022). Foliar Application of Different Iron Sources Improves Morpho-Physiological Traits and Nutritional Quality of Broad Bean Grown in Sandy Soil. Plants.

[53] Al-Amiri M.S. (2013). Improved growth, productivity and quality of tomato (*Solanum lycopersicum* L.) plants through application of shikimic acid. Saudi J. Biol. Sci..

[54] Zhao S., Zhou N., Zhao Z.-Y., Zhang K., Wu G.-H., Tian C.-Y. (2016). Isolation of endophytic plant growth-promoting bacteria associated with the halophyte *Salicornia europaea* and evaluation of their promoting activity under salt stress. Curr. Microbiol..

[55] Lee J.M., Oda M. (2003). Grafting of herbaceous vegetable and ornamental crops. Hortic. Rev..

[56] Hassan A.A. (2008). Tomato (in Arabic).

[57] Khan M.A., Asaf S., Khan A.L., Adhikari A., Jan R., Ali S., Imran M., Kim K.M., Lee I.J. (2019). Halotolerant Rhizobacterial Strains Mitigate the Adverse Effects of NaCl Stress in Soybean Seedlings. Biomed Res. Int..

[58] Helrich K. (1990). Official Methods of Analysis.

[59] Katoch R. (2011). Analytical Techniques in Biochemistry and Molecular Biology.

[60] Moran R. (1982). Formulae for determination of chlorophyllous pigments extracted with N, Ndimethylformamide. Plant Physiol..

[61] Sharma S.K., Le Maguer M. (1996). Lycopene in tomatoes and tomato pulp fractions. Ital. J. Food Sci..

[62] Singleton V.L., Rossi J.A. (1965). Colorimetry of total phenolics with phosphomolybdic phosphotungstic acid reagents. Am. J. Enol. Vitic..

[63] Jackson M.L. (1973). Soil Chemical Analysis.

[64] Polle A., Otter T., Mehne-Jakobs B. (1994). Effect of magnesium deficiency on antioxidative systems in needles of Norway spruce [*Picea abies* (L.) Karst.] grown with different ratios of nitrate and ammonium as nitrogen souices. New Phytol..

[65] Giannopolitis C.N., Ries S.K. (1977). Superoxide dismutases: Occurrence in higher plants. Plant Physiol..

[66] Beyer W.F., Fridovich I. (1987). Assaying for superoxide dismutase activity: Some large consequences of minor changes in conditions. Anal. Biochem..

[67] Iturbe-Ormaetxe I., Escuredo P.R., Arrese-Igor C., Becana M. (1998). Oxidative damage in pea plants exposed to water deWcit or paraquat. Plant Physiol..

[68] Fales T.M., Jaouni J.F., Babashak I. (1973). Simple device for preparing ethereal diazomethane without resorting to codistillation. Ann. Chem..

[69] AOAC (Association of Official Analytical Chemists) (1990). Official Methods of Analysis.

[70] Mohammed I.A., Guma A.N. (2015). Anatomical diversity among certain genera of Family Cucurbitaceae. IJRSB.

[71] Gomez K.N., Gomez A.A. (1984). Statistical Procedures for Agricultural Research.

[72] Snedecor G.W., Cochran W.G. (1980). Statistical Methods.

